# Recent developments in targeting access to high cost medicines in Australia

**DOI:** 10.1186/1743-8462-2-28

**Published:** 2005-11-23

**Authors:** Christine Y Lu, Jan Ritchie, Kenneth M Williams, Richard O Day

**Affiliations:** 1 School of Medical Sciences, University of New South Wales, Sydney, Australia; 2 Department of Clinical Pharmacology and Toxicology, St Vincent's Hospital, Sydney, Australia; 3 School of Public Health and Community Medicine, University of New South Wales, Sydney, Australia

**Keywords:** Access to medicines, tumour necrosis factor inhibitors, pharmaceutical benefits scheme, drug reimbursement

## Abstract

**Background:**

In Australia, the Pharmaceutical Benefits Scheme (PBS) has developed a set of arrangements to control access to high-cost medicines to ensure their use is cost-effective. These medicines include the tumour necrosis factor-alpha inhibitors (TNFIs) for the treatment of rheumatoid arthritis. The aim of this first phase of a qualitative study was to explore basic views on the restricted access to TNFIs in order to confirm where further investigation should take place in the next phase.

**Methods:**

Semi-structured interviews were conducted in 2004 with a member of the four relevant stakeholder groups. Participants were asked their opinions about features of the establishment, process and effects of the system of restricted access to TNFIs. Views on the collaboration between stakeholder groups in the decision-making process were also collected.

**Results:**

The principle of 'controlled access' to TNFIs was supported in general. There were concerns regarding some of the specific eligibility criteria. Wider and more transparent stakeholder consultation was judged desirable. Some flexibility around prescribing of TNFIs by physicians, and regular review of the arrangements were proposed. These themes will inform the next phase of the study.

**Conclusion:**

This first phase highlighted a range of issues associated with the PBS arrangements restricting access to TNFIs. Timely review and report of issues and concerns associated with such policy developments that arose in practice are essential. There is a need for a more comprehensive exploration across a wide range of stakeholders with different perspectives that will in turn be helpful in guiding policy and practice around national arrangements to manage access to high-cost medicines.

## Background

Expenditure on pharmaceuticals has increased dramatically in all countries both in the developed and the developing world in recent years [1]. Provision of an increasing number of effective but expensive pharmaceuticals by drug reimbursement systems is challenging because the costs per patient are high. Payers in the public and private health systems face similar challenges due to increasing consumer expectations to access these medicines in the context of substantive cost constraints. The Pharmaceutical Benefits Scheme (PBS) is Australia's national program which provides subsidised access to prescriptions medicines for the community [2]. Decisions on drug reimbursement ('listing') under the PBS are based on assessment by the Pharmaceutical Benefits Advisory Committee (PBAC), which evaluates efficacy, safety and incremental cost-effectiveness of new pharmaceutical products compared to other existing treatments [3]. Australia was the first country to introduce an explicit requirement for economic analysis to select pharmaceuticals for a publicly funded formulary [4]. This system has attracted considerable attention worldwide as Australian pharmaceutical prices are markedly lower than those in other comparable countries [5]. The national goal, as expressed in Australia's National Medicines Policy and, in particular, the Quality Use of Medicines component, states that limited resources should be utilised in such a way that there is provision of needed, effective and safe medicines that are affordable for the individual in order to achieve optimal health outcomes [6].

Biological agents licensed in Australia for treating rheumatoid arthritis include three tumour necrosis factor-alpha inhibitors (TNFIs), etanercept (Enbrel^®^), infliximab (Remicade^®^), and adalimumab (Humira^®^), and an interleukin-1 receptor antagonist, anakinra (Kineret^®^). These new medicines markedly reduce disease activity in a majority of patients, but there are concerns about their high cost (approximately AUD$20,000 per patient per year), in particular given the annual growth in government expenditure on pharmaceuticals averaging 10.5% between 1992–93 and 2002–03 (representing an increase from AUD$1.88 billion to AUD$5.12 billion) [7]. Further, there are uncertainties regarding their long-term safety, including serious and opportunistic infections and the risk of lymphoma [8,9].

The PBS has evolved a set of arrangements to control access to high-cost medicines in an attempt to maintain the viability of the PBS. Representative, but most innovative of this set of arrangements are those established for the TNFIs, implemented since August 2003. The decision to subsidise TNFIs was based on a collaborative decision-making model to enable the listing of the TNFIs on the PBS. This involved the PBAC which consulted the relevant key stakeholders: the respective pharmaceutical companies, and rheumatologists via the Australian Rheumatology Association Therapeutics Subcommittee. The arrangements for access under the PBS (PBS-restrictions), formulated to secure the most cost-effective use of these expensive agents, were agreed upon after extensive negotiations [3,10]. The respective consumer organisation, the Arthritis Foundation of Australia, also coordinated a strong lobbying effort by consumer representatives to the government. This collaborative model has set a new paradigm for future PBS decisions. Subsidised access was limited to a subset of patients whose disease "has not been adequately controlled using conventional anti-rheumatic drugs and these patients must meet strict criteria for both starting and continuing biological therapy" [11]. Patients are required to sign a Patient Acknowledgement Form which specifies that continuation of therapy beyond four months "will only be approved if objective, substantial response is achieved". Prescribing rights are limited to specialists with expertise in the management of rheumatoid arthritis. Also agreed upon was a risk-mitigation arrangement between the government and the sponsors that established a ceiling level for government outlays annually [10]. Similarly complex arrangements for access are being applied to other high-cost medicines such as imatinib (Glivec^®^) for the treatment of patients with chronic myeloid leukaemia [11].

Decision makers' and stakeholders' perspectives on prioritising decisions about drug reimbursement and decision-making processes in comparable countries such as Finland, Canada, and the United Kingdom have been described [12-14]. However, to the best of our knowledge, there are no published data on perceptions regarding approaches used in Australia, such as arrangements for access to high-cost medicines operated through the PBS, or studies examining stakeholder consultation processes to reach consensus on arrangements for subsidised access to high-cost medicines. We considered that careful examination of the recent developments in targeting access to high-cost medicines, using TNFIs as an example, would be instructive in informing the debate concerning the principles and processes that might underpin appropriate and ethical access to expensive pharmaceuticals under the PBS or similar access systems. Stakeholders are defined here as groups of people that have the potential to influence the decisions on the arrangements for access, or those who are affected by the PBS-restrictions. The views, attitudes, concerns, and level of support for these arrangements by the stakeholders are critical determinants for a successful implementation of the arrangements. Qualitative techniques are useful to explore perceptions and experiences across a range of relevant stakeholders with respect to restricted access to TNFIs, and to understand the effects of the access criteria when implemented in practice. From a policy perspective, issues and concerns that arise in practice need to be reviewed in a timely manner thereby enabling appropriate management of any implications. Research guiding policy development can often best be undertaken as an iterative approach with each phase building on the one before, in particular where the impact of implementing new access criteria on practice is unknown. The scope and content of such a study are not specified in advance but are developed iteratively. This paper reports on the initial phase of a qualitative study using an iterative approach. This first phase sought to explore the views of one member of each of the four stakeholder groups with a vested interest in any new policy about provision of medicines, in order to confirm where further exploration should take place in the next iteration.

## Methods

Qualitative techniques were used for data collection and analysis. In depth, semi-structured interviews were conducted in Sydney in 2004 with four individuals to explore four different perspectives about access to TNFIs under the PBS. Interviewees were asked their opinions about features of the establishment, process and effects of the arrangements for targeting access to TNFIs. Views on the decision-making process and collaboration between stakeholder groups were also collected.

Due to a finite number of key stakeholder representatives who participated in the PBAC decision-making process that formulated the arrangements for access to TNFIs, they were purposively *not *invited in this initial phase of the study. Purposeful sampling was used to select participants on the basis of their primary membership of different stakeholder groups with respect to the controlled access to TNFIs: a rheumatologist, a health advisor to the government, an employee of a pharmaceutical company, and a patient (who had used a TNFI). Representation of participants was not sought in this first phase of the study which aims to elicit a basic viewpoint from a member of each stakeholder group. The rheumatologist received an invitation to participate through an opinion-leader rheumatologist, purposively selected to have had experiences prescribing TNFIs through the PBS at the time of the study. The health advisor who participated belonged to a like-group of the PBAC, and the pharmaceutical employee was invited to give views of the industry but was not from a company that marketed TNFIs. The patient was nominated by a rheumatologist. Participant information sheets and consent forms were provided to all participants prior to the interviews. These four interviews were conducted by one trained researcher (CL); interviews were of 45 to 60 minutes duration.

Interviews were recorded and transcribed verbatim. The transcripts were coded for major concepts. Categories related to the discussion topics were created. Coded segments were then analysed and categorised thematically, thereby developing a thematic framework. NVivo software 2.0 was used to help manage the qualitative data. The transcripts were also coded independently by another investigator (RD or KW). The researchers then met to examine the analyses in order to reach agreement on categories and identified themes. Some differences with regard to labelling the themes were found, but agreement was reached on the central meanings. Interviewees were offered opportunities to review an edited interview transcript to check that their views had been accurately represented. Only one interviewee reviewed the transcript. It was accepted without change. The study was approved by the Human Research Ethics Committees of St Vincent's Hospital Sydney, and the University of New South Wales, Australia.

## Results and Discussion

This initial phase identified a range of concerns, as held by individuals from different stakeholder groups, that arose in practice associated with the recent developments to control access to a group of high-cost medicines in Australia. These are imperative issues that decision makers in Australia and other countries need to consider and manage appropriately and in a timely manner. The pre-determined interview topics and major themes that emerged from the data are listed in Table 1. The four major themes are described below with selected quotes from participants.

**Table 1:** Major themes around restricted access to tumour necrosis factor inhibitors

**Predetermined Interview topics**
Perceptions of PBS-restrictions on access
Experience with application process
Pre-PBS collaboration between the different stakeholder groups
Post-PBS collaboration between the different stakeholder groups
Sources of information

**Major themes emerging from data**
Access to medicines
Targeting access
Review of the PBS-restrictions
Implications of access arrangements in practice
Effects on the practice of rheumatology
Ethical issues
Roles and responsibilities
PBS Decision-making process
Stakeholder consultation process
Transparency
Information provision

### 1. Access to medicines

#### 1.1 Targeting access

Interviewees expressed an understanding for the finite resources that can be allocated to treat the possible range of diseases. The principle of 'controlled access' to TNFIs was supported in general. All perceived that the purpose of restricting access to TNFIs was to control costs while targeting those patients identified by the access criteria as most likely to derive benefit.

"The government is trying to target the therapy to those who need it ... and control their budget." (advisor)

The PBS-restrictions on access, including the limiting of prescribing rights to qualified specialists, were considered to be safe and cost-effective, generally evidence-based and appropriate, given the expense and some lingering uncertainty about long-term safety.

"TNF inhibitors are fairly new ... there are no long-term data as yet on their use ... they are not without side effects, and because of their expense, trying existing therapies first is a reasonable approach" (rheumatologist)

Participants disagreed with some of the specific eligibility criteria which they interpreted as inflexible and not obviously evidence-based. For example, a number of patients with severe rheumatoid arthritis suffered because of the requirement that rheumatoid factor be positive.

"The requirement for a rheumatoid factor is a concern because I don't believe that there's any scientific evidence ... and they [application assessors] seem to be inflexible about that criterion ... There are unfortunate people who would qualify on every criteria except rheumatoid factor alone." (rheumatologist)

#### 1.2 Review of the PBS-restrictions

Availability of TNFIs through the PBS, although tightly restricted, was welcomed. However, ongoing review of the PBS-restrictions in accordance with emerging new evidence and analysis of utilisation data was seen as both important and necessary. Interviewees believed the key stakeholder representatives who had participated in the initial stakeholder consultation should again participate in these activities. The risk-mitigation agreement should also be reviewed for its effectiveness and value.

"There should be some process of review of criteria, after some agreed period, particularly if they're sharing a [financial] risk, to see whether they still continue as appropriate based on new evidence and data of utilisation, etcetera." (advisor)

### 2. Implications of access arrangements in practice

#### 2.1 Effects on the practice of rheumatology

The arrangements for access to TNFIs requiring evidence of exposure to and failure of a number of anti-rheumatic drugs appear to have promoted re-evaluation of previous treatments and responses in patients with insufficiently controlled rheumatoid arthritis. A proportion of patients may have benefited from this process with better control of their disease without reaching the 'disease activity' specified in the eligibility criteria.

"It's forced our rheumatologists to re-look at the treatment that patients have already received and to re-evaluate ... in reassessing the patient and aiming to meet the PBS criteria, they actually get their rheumatoid arthritis under control before requiring a biological..." (rheumatologist)

#### 2.2 Ethical issues

One of the ethical issues raised by the interviewees was the possible benefit that might be achieved in some patients if the PBS-restrictions would allow a switch to another TNFI if they failed to respond to the first agent.

"If patients didn't meet the criteria for continuing treatment and at the time they stopped, were not bad enough to meet the initial criteria for another agent, they can't switch to another agent, because they are required to meet the initial criteria again." (rheumatologist).

The original PBS-restrictions did not address this issue, but these have been altered shortly after the completion of this study's initial phase. The PBS introduced an 'interchangeability' rule from December 2004, allowing patients who meet the eligibility criteria for commencing therapy to trial any of the listed TNFIs as well as anakinra, another anti-rheumatic biological agent [11]. This change demonstrates that some review of the PBS-restrictions is occurring.

Despite the 'objective' clinical measures upon which continued access to TNFIs is determined, there are many factors influencing the medical condition of an individual. Concerns were raised about withdrawing patients from an effective treatment when they may have just missed the threshold for continued access as well as a lack of flexibility for physicians to make decisions in cases that are equivocal based on their professional judgement.

"There's a disparity between the patients' clinical response and joint count and their laboratory parameters ... the inflammatory markers may be staying up when they actually clinically are a lot better." (rheumatologist)

"There's got to be a level of discretion there to the experts because they're the guys who know. It's not some bureaucrat." (patient)

The application process was seen to be complex, time-consuming and an administrative burden for everyone involved:

"The administrative burden for the doctors, for the Health Insurance Commission...on the pharmacists, and also the patients...and the whole being caught up in that bureaucratic tangle." (pharmaceutical employee)

Another ethical concern was that a Patient Information Sheet had not been developed to accompany the Patient Acknowledgement Form at the time these interviews took place, i.e. 12 months after the first TNFI was made available under the PBS.

"They're [the patients] signing a contract and they should be able to understand the benefits and also the implications...they need to do it in a manner where they have all the information they need to decide." (advisor)

#### 2.3 Roles and responsibilities

Broadly, government is expected to provide affordable access to effective medicines for patients with medical needs while using taxpayers' money wisely and appropriately. A related and necessary responsibility of government is to monitor the PBAC process to ensure appropriate assessment and selection of medicines for reimbursement by the PBS.

"The government has an obligation to ensure that the processes that the PBAC uses are rigorous, are regularly evaluated, and are well supported." (advisor)

The pharmaceutical industry interviewee felt that information provision by industry was a major responsibility and needed particularly when high-cost medicines subject to complex eligibility criteria become available through the PBS. This interviewee considered that updates should be provided to prescribers about the clinical uses of medicines, including the application procedures for PBS-subsidised treatment; and to promote marketing and publicity with the content being concordant with the PBS-restrictions (A consequence of risk-mitigation agreements is a strong negative incentive for inappropriate marketing and publicity leading to use outside approved PBS-restrictions). The risk-mitigation agreement that is part of the arrangements for access to TNFIs is an indication of collaboration between the government and the pharmaceutical company.

Physicians have a responsibility to comply with the PBS-restrictions by law [15]. The PBS-restrictions were worded in such stringent and exhaustive detail that they were seen to demonstrate a lack of faith in the prescribers by the PBS.

"There seems ... mistrust from the PBS that rheumatologists could have a loophole for the prescribing ... that they're worried that we will be prescribing the drug for patients who don't strictly have rheumatoid arthritis." (rheumatologist)

A view was put that if physicians disagree with any of the restrictions, they should lobby through their professional organisation for revision because they are ultimately responsible for their patients' well-being. More reliance on physicians was considered appropriate and desirable.

"Prescribers are obliged to adhere to the PBS arrangements... If we don't like the criteria and have concerns with it then the appropriate thing is to lobby through the ARA [Australian Rheumatology Association]..." (rheumatologist)

Concerns were voiced about the need to achieve substantial clinical improvement for continuing access at the follow-up assessment. This requirement may have introduced potential perverse incentives to 'fudge' measurements of disease activity in order to ensure continued access to TNFIs. This raises the issue of the responsibility and accountability of prescribers with respect to their legal obligation to comply with the PBS requirements for PBS-subsidised treatments. However, this does place powerful ethical concerns on doctors in cases where they become the agents whereby patients are denied or withdrawn from effective medicines, particularly if the patient's response is near the threshold level of eligibility. The Australia Rheumatology Association was seen to be responsible for reviewing the PBS-restrictions and providing information to their members, i.e. rheumatologists, as well as the patients.

"The ARA [Australia Rheumatology Association] has an obligation to provide information to their own members, it's essential that they support with adequate provision of education and advice and support about this new treatment ... and they also have a role to provide education to the public." (rheumatologist)

The role and responsibilities of patient organisations were not explored in detail in this initial phase. Consumer organisations could represent patients in the consultation processes, but qualifications of the staff working in such organisations were thought to be important if they are to contribute to the consultation processes or to produce educational information. Consumer organisations were perceived to primarily interact with patients and to disseminate information.

"It's difficult to allocate the Arthritis Foundation a more active role in making recommendations, depending on who is actually staffing the Arthritis Foundation and where those opinions are coming from...the Arthritis Foundation is primarily interfacing with the patients." (rheumatologist)

The staff at the Health Insurance Commission, a statutory organisation administering the PBS and other health programs [16], was considered in general to be helpful with respect to queries regarding the application procedure for subsidised TNFI treatments.

### 3. PBS Decision-making process

#### 3.1 Stakeholder consultation process

The interviewees were in substantive accord with the initiative instituted with TNFIs, that negotiations between the stakeholders should take place early in the decision-making process to enhance the likelihood that the final arrangements for access to such medicines are reasonable and can be supported.

"Negotiations should start early so that there is clear communication between the expectation and the calibre of evidence needed." (advisor)

"...so that the final criteria are the correct criteria, the right patient group is being identified, and also the initiation and continuation rules, etcetera, are workable and manageable" (pharmaceutical employee)

However, the interviewees had different views about the stakeholder consultation process that had taken place.

"The major strength is the negotiations and the partnering." (advisor)

"It seems that each group has their own agenda and it's not really like a collaboration but more each trying to put forward their opinion." (rheumatologist)

While the representativeness of the Australian Rheumatology Association Therapeutics Sub-committee in the decision-making process was believed to be appropriate, a wider consultation among ordinary members of the association was wanted:

"The therapeutics committee should have sought information from the members of the ARA [Australian Rheumatology Association], or have consulted more widely before they made their decisions because it seems that they made their decisions on our behalf but without consulting us." (rheumatologist)

On the subject of representing the views of patients in such processes, the rheumatologist was of the opinion that doctors would appropriately represent the views of their patients. On the contrary, the three other participants felt that patients should have a more direct role in the process, which could provide a humane view; and patients should at least be involved in the development of the Patient Acknowledgement Form.

"No one really understands what you go through except you ... not even my rheumatologist or other specialist." (patient)

"Patient groups should have a seat at the table...they should be represented formally, rather than by the doctors... We need to have a process where consumers, prescribers, people with interest, come together on an equal footing to discuss what the issues are and what the criteria are and can follow the progress." (advisor)

There is increasing need for the voices of patients, their carers and the public to be heard because it is appropriate and crucial for acceptance, change and improvement in the process [17-19]. The representativeness of some consumer groups was raised along with concern that some are funded in part by pharmaceutical companies. This concern may be reduced if conflicts of interests are declared openly, which links to the theme of "transparency".

#### 3.2 Transparency

Transparency of the PBAC process and their decisions was a recurrent theme in the interviews. Interviewees thought that wider involvement in the consultation process and greater empowerment of stakeholders to contribute to discussion of the issues would be desirable. Disclosure of the rationale behind the PBAC's selection of access criteria, currently not available in the public domain, was considered to be desirable and a helpful improvement to the system. Greater empowerment (and transparency) by provision of appropriate information was proposed.

"A public document which summarised why different decisions were made, and the evidence base on which they were made would be a most fruitful thing to help patients to understand why they have potentially limited access to some of these medicines ... that's about empowerment in information." (advisor)

In addition, the issue of the requirement for a positive rheumatoid factor has been the subject of debate since the PBS-restrictions for TNFIs were implemented [20,21]. Greater transparency would be helpful to resolve these concerns and gain increased community support. The recent recommendation by PBAC to remove this criterion [22] is a welcome and rational outcome of the lengthy debate on this issue.

The interviewees who participated in this study were 'outsiders' to the consultation process that led to establishment of the arrangements for access. Minimal involvement in the decision-making process and the current level of transparency around PBAC's decisions limited their support for some of the eligibility criteria. Further, their minimal awareness of the negotiations that took place between key stakeholders suggests that there is a need for better communication between those key stakeholder representatives with their constituencies. Increased communication within and between the stakeholder groups is also critical to obtain stronger support for the final PBS-restrictions in practice.

### 4. Information provision

The PBS-restrictions to control access to high-cost medicines, exemplified by TNFIs, are particularly complex, and thus adequate provision of information is all the more important. Information from pharmaceutical companies to the prescribers and general practitioners were noted to be abundant, however, interviewees considered that the content needed to be more balanced. In particular, the rheumatologist and the government advisor expressed a lack of faith in the pharmaceutical companies' ability to supply appropriate materials for patients or the public.

"It is not the sponsor's responsibility to talk to patients because they will always get it wrong... direct-to-consumer advertising is a crazy way to go..." (advisor)

Some concern was also expressed about industry-funded 'educational' functions:

"The way it was presented was one-sided, that the government has put all these barriers forward, the barrier nature was highlighted..." (advisor)

Interviewees' had concerns about the understanding of the PBS system and the PBAC process among health professionals and in the community. Better provision of information on the structure of the PBS and the PBAC process was proposed. In particular, interviewees noted that doctors are the most appropriate health professionals to inform patients with regards to the PBS-restrictions, and that they therefore need to be better informed about the cost-effective analyses that underpin the PBAC decisions. For example, patients should be made aware that self-funding is an option, although in the case of TNFIs this is impractical for most people. Interviewees proposed that communication about the need to targeting use of highly specialised medicines should extend to (potential) patients and the public generally, and also to general practitioners who could have an important role and contribution in this area. Increased community awareness and understanding of the principle of quality-use-of-medicines should be part of the overall educational strategy.

Interviewees had different views on whose responsibility it was to provide information. The rheumatologist considered that information about the efficacy and safety of TNFIs is most important and that the Australian Rheumatology Association would be appropriate to provide ongoing updates. Other proposed providers of information: pharmacists, the  Medicare Australia (previously the Health Insurance Commission), the PBS, the National Prescribing Service (an independent educational organisation funded by the Federal Government) [23], and the consumer organisations.

### Further investigation

The initial phase of the study has explored concerns of individuals from the four relevant stakeholder groups. Our study was not designed to support statistical generalisability. Findings have established the basis for the next phase of the study to explore perceptions of a wide range of stakeholders that will be helpful in gaining a comprehensive and broad societal view of these significant developments of controlling access to high-cost medicines. A written interview guide has been developed on the basis of the findings of this phase for use in subsequent interviews. Diversity in sample characteristics and inclusion of individuals who were engaged in the decision-making process will expand understanding of the issues affecting different members of the stakeholder groups and provide some insight into the negotiations during the decision-making process. Interviewees will include government advisors, participants in the stakeholder consultation process that formulated the arrangements for access to TNFIs (who are in a position to comment on what actually occurred and what could be improved), rheumatologists and patients who have gone through the application process, pharmaceutical company personnel, consumer organisation representatives, and government administration staff. We anticipate that this broad range of interviewees will provide deep and rich insights to decision makers regarding the future decision-making process and development of national arrangements to manage access to medicines from the perspectives of enhancing appropriate access to effective and expensive medicines.

## Conclusion

The PBS has developed a set of arrangements to target access to expensive medicines such as the TNFIs in an attempt to sustain this national drug reimbursement system. Findings from this initial phase of the study highlighted a number of issues associated with the restricted access to TNFIs. The principle of 'controlled access' to TNFIs was supported in general by all interviewees each from a different stakeholder group. However, it is clear that some views, such as those concerning patient direct participation or the varying responsibilities of each stakeholder group, were different. The pre-determined questions appeared to adequately explore the key issues. A thematic framework has now been developed on the basis of the major themes that emerged from the interviews. We believe there is a need for a more comprehensive exploration, through a second phase of the study, to elicit different perspectives across a variety of stakeholders, that will in turn be helpful in guiding policy and practice around national arrangements to manage access to high-cost medicines.

Australia's Pharmaceutical Benefits Scheme, as part of the National Medicines Policy, is committed to providing access to cost-effective medicines to the community. When resources are constrained, restricting access is an inevitable outcome. Greater and more trust-based communication and discussion among a wide range of stakeholder groups will enhance community awareness and support to limit access to effective medicines. Acceptable but different approaches to control access and manage the associated implications should be explored with the goal of enhancing health outcomes for all.

## Declaration of competing interests

Christine Lu and A/Prof Jan Ritchie have nothing to declare. A/Prof Ken Williams is a member of the Advisory Board to the sponsor for adalimumab. Prof Ric Day is a member of the Advisory Board to sponsors for adalimumab, infliximab, and anakinra in Australia. A/Prof Williams and Prof Day have also been contracted to undertake clinical trials with etanercept, infliximab, adalimumab, and anakinra. Recompense for these activities received was placed in audited hospital trust funds for use in the research activities of the Clinical Pharmacology Department, St Vincent's Hospital, Sydney.

## Authors' contributions

CL, KW, and RD have made substantial contributions to the conception and design of the study, analysis and interpretation of the data; and drafting and revising the manuscript. JR has been involved in advising on the analysis, drafting the manuscript and revising it critically for intellectual content. All authors read and approved the final manuscript.
